# Real-Time Tracking Framework with Adaptive Features and Constrained Labels

**DOI:** 10.3390/s16091449

**Published:** 2016-09-08

**Authors:** Daqun Li, Tingfa Xu, Shuoyang Chen, Jizhou Zhang, Shenwang Jiang

**Affiliations:** 1 School of Optoelectronics, Image Engineering & Video Technology Lab, Beijing Institute of Technology, Beijing 100081, China; 2120140539@bit.edu.cn (D.L.); cccyy0713@163.com (S.C.); xiaomianzhou@126.com (J.Z.); jiangwenj02@163.com (S.J.); 2 Key Laboratory of Photoelectronic Imaging Technology and System, Ministry of Education of China, Beijing 100081, China

**Keywords:** real-time tracking framework, Forward–Backward error, ensemble classifier, location constraint, embedded system

## Abstract

This paper proposes a novel tracking framework with adaptive features and constrained labels (AFCL) to handle illumination variation, occlusion and appearance changes caused by the variation of positions. The novel ensemble classifier, including the Forward–Backward error and the location constraint is applied, to get the precise coordinates of the promising bounding boxes. The Forward–Backward error can enhance the adaptation and accuracy of the binary features, whereas the location constraint can overcome the label noise to a certain degree. We use the combiner which can evaluate the online templates and the outputs of the classifier to accommodate the complex situation. Evaluation of the widely used tracking benchmark shows that the proposed framework can significantly improve the tracking accuracy, and thus reduce the processing time. The proposed framework has been tested and implemented on the embedded system using TMS320C6416 and Cyclone Ⅲ kernel processors. The outputs show that achievable and satisfying results can be obtained.

## 1. Introduction

As one of the fundamental research topics in military spying, security surveillance and smart controlling to build “smart city” or “safe city”, object tracking has been playing an important role in many applications. However, due to the variety of the object appearance, the motion of the object, the complexity of the motion background such as the occlusion, designing real-time tracking system still poses challenges to academia.

Adaptive tracking-by-detection method has been popular in recent years and researchers have made great efforts in the analysis algorithm to overcome the challenge [1,2,3,4,5,6]. In recent years, more and more algorithms treat the tracking problem as the classification task which regards the object tracking as a binary classification issue.

The randomized forest classifier has become one of the most valuable ways for classification because of its high speed, high accuracy and the possibility of incremental update. It is a tree-based ensemble classifier with each base classifier depending on a collection of random variables [7]. Most algorithms [8,9,10] construct the base classifier with a set of pixel comparisons, which are generated offline at random and remain unchanged in run time. By using such a simple approach, not only the cost of the computation could be decreased, but also memory space could be saved. However, when the patches to be trained are sufficiently large, those basic trees cannot be applied due to the cost limitation.

To address these issues, we use a novel tracking framework with adaptive features and constrained labels (AFCL) to reduce the above limitations.

### 1.1. Motivations

Aiming at improving the efficiency when the training set is large, papers [9,11] use a number of ferns as the classifier and adopt the two bit Binary Patterns [11,12] to get the feature vectors. In [9], the theoretical comparison between the ferns and the random trees is conducted, and the results show that the ferns perform much better than the trees when the training set is large. Because of the multiplicative behavior, ferns have stronger discriminating power under complex circumstances. Although the tracking effect is improved by using ferns, the features are not stable in runtime. If the object has changeable appearance with affine variation, or the noise of illumination in the background becomes abrupt, feature vectors may lose their reliance.

In addition, we find that learning methods such as the P-N learning [12], multiple-instance learning [13,14] and semi-supervised learning [15,16] applied by current state-of-the-art algorithms decrease the noise in some degree, but they are not solving the real problem. Classifier used in previous studies is trained only with binary labels without the information of the transformations. The so-called positive samples and negative samples are selected by artificial process. The positive samples are adjacent to the current object location, and the negative samples are away from the object center [11,12,17], which involves the label noise as well as follow-up tracking conditions. To address these issues, we attempt to present a novel tracking framework (AFCL).

### 1.2. Contributions

The contribution of the present study can be summarized as follows:

(1) The feature vectors are improved. We adopt the Forward–Backward error [18] to update the pixel pairs. In this way, each fern can be adaptive in runtime and effectively avoid the deviation caused by object noise. In this paper, we adopt the ferns as the base classifier, and each fern is based on a set of pixel comparisons [11]. We take advantage of the pixel pairs which are used as comparison in the last frame, recording their information and predicting the locations in the next frame by using Lucas–Kanade tracking [19].
(2) The label noise is decreased. To divide the samples more objectively and import the information of the samples’ transformations, a novel location constraint comes up. It is a Gaussian fashion function, which can assign the weight to each sample according to the location. We can change the amount of the samples and label them in runtime by controlling the scaling factors. Therefore, all the positive or negative samples can be treated as uncoordinated samples according to their weights.
(3) Adopt the combiner to assist learning. After filtering the patches by the ensemble classifier, we have several bounding boxes left that are supposed to be included in the target. To reach the target, we adopt the combiner to evaluate the most valuable bounding box, and regard it as the target to train the classifier in current frame. Firstly, we transmit the posterior probability of each box into the combiner. Secondly, we match the box with the compressed templates by using normalized cross-correlation (NCC). Finally, by combining the outputs from the NCC, the posterior probability and the value from location-weighted function, we ensure that the bounding box includes the target in real time.

The rest of the paper is organized as follows: In Section 2, we introduce the AFCL framework in detail. The embedded system with TMS320C6416 and Cyclone Ⅲ as the kernel processors are introduced in Section 3. In Section 4, we analyze the comparative experiments of the framework and the experiments of the embedded system. In Section 5, we reach the conclusions of the paper.

## 2. AFCL Framework

**Figure 1:**
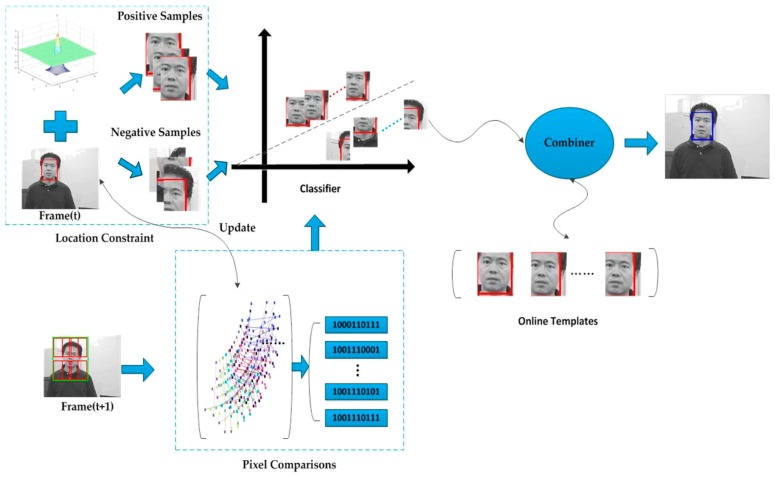
Flow chart of the AFCL framework.

Figure 1 presents a detailed flow chart of the AFCL framework. This framework firstly uses the location constraint and the target in Frame (t) to get the positive and negative samples, after which it trains the classifier with these samples. At the same time, the pixel comparisons in Frame (t) are utilized, and Forward–Backward error and Lucas–Kanade (L–K) tracking are used to update the pixel comparisons in the next frame. When the Frame (t + 1) arrives, all the bounding boxes are generated around the target that had been found in the last frame. The updated pixel comparisons are also used to derive the feature vectors, which are then transmitted to the classifier. After the classification, the bounding boxes that cannot be confirmed are received by the combiner, and evaluated by the online templates. Finally, the most valuable box is chosen as the target. If its value is greater than the threshold value, the box will be sent to the classifier and update the templates. The crucial procedures in our framework are further clarified in Section 2.1, Section 2.2 and Section 2.3.

### 2.1. Adaptive Features

Pixel comparisons are always generated offline and kept fixed during runtime [8,9,10,11]. When there exists illumination variation or rotation, it is inevitable that the fixed pixel comparisons bring errors, which not only results in inaccurate features, but also exerts negative influences on the classifier. Therefore, it is necessary that adaptive comparisons are made during run-time. The AFCL framework adopts the Forward–Backward error to evaluate and update the points, which are used as pixel comparisons in real-time. It can keep the features adaptive and thus make the detection more accurate.

#### 2.1.1. Forward–Backward Error

In the Forward–Backward (F–B) method, the error is used in real-time to evaluate the tracking. By using the L–K tracking forward and backward method, a sparse motion flow is generated between Frame (t) and Frame (t + 1), and each point is assigned an error [18].

**Figure 2:**
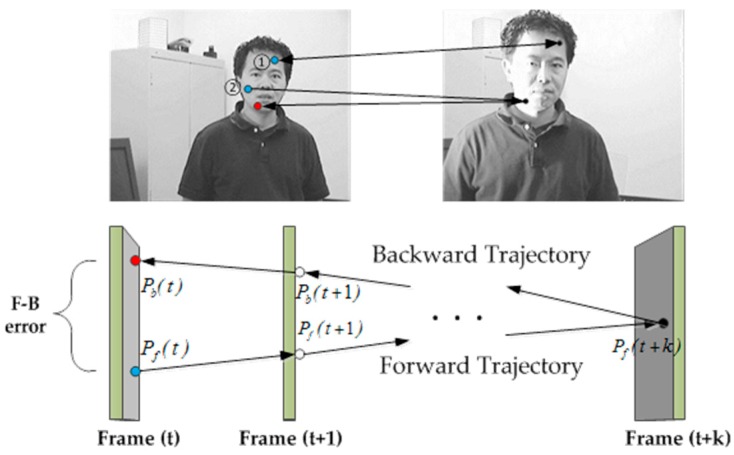
Illustration of the Forward–Backward error.

The F–B error is shown in Figure 2. From the figure (top), we can easily derive the method to evaluate the value of the specific point. Point 1 keeps the gray value in both images. When tracking this point by L–K tracking forward or backward, we will have the same trajectories. The location can also be calculated correctly in real-time. However, when tracking Point 2, the result is different from expectation. From the two images, we can easily find that the illumination of the target has changed in the right one. Thus, the tracker cannot localize the point accurately, which results in a wrong position when tracking forward. Tracking this point backward will generate a location that is different from the original. We can thus utilize this inconsistency to identify whether the point needs to be updated. 

Here, we let F={Fame (t),Frame (t + 1),...,Frame (t +k)} be the image sequence and $P_{f}(t)$ be the point location when tracking forward in time t, $P_{b}(t)$ be the location when tracking backward. By using the L–K tracker, the point $P_{f}(t)$ will be tracked forward for k steps. The trajectory can be defined as $T_{f}=\{P_{f}(t),P_{f}(t+1),...,P_{f}(t+k)\}$. Similarly, the trajectory which is used to track backward can be defined as $T_{b}=\{P_{b}(t),P_{b}(t+1),...,P_{b}(t+k)\}$ where $P_{b}(t+k)=P_{f}(t+k)$. Thus, we can use the distance between these two trajectories to define the Forward–Backward error. Several distances can be used to compare trajectories. In this paper, we use the Euclidean distance between the original point and the end point for error evaluation.

#### 2.1.2. Adaptive Features

Keeping the features adaptive and accurate can make the tracking more robust and stable. In this subsection, we will introduce how one can generate these adaptive features.

**Figure 3:**
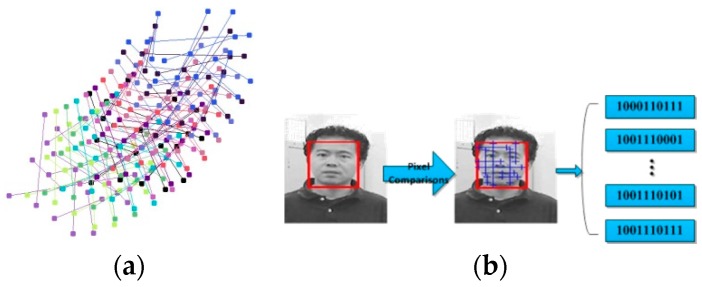
Pixel comparisons and the generation of the feature vectors: (**a**) pixel comparisons; and (**b**) the generation of the feature vectors.

At the very beginning, we generate all the points used as pixel comparisons randomly (see Figure 3a) and store the locations of each point offline. We treat ten comparisons as one fern and therefore construct ten ferns in total. To ensure the independence of each fern, the comparisons are split randomly. Thus, each fern is constructed by different pixel comparisons and all the ferns together cover the patch which is not yet detected. When one frame arrives, the pre-populated sets of pixel comparisons are stretched to the patch. Each fern can return the value 0 or 1 and generate one binary feature vector in real-time as shown in Figure 3b.

When a new frame comes, we can utilize the coordinate of each point in the last frame and the L–K tracking to get the new position in the current frame. The quality of the point can be evaluated by using F–B error. If its F–B error goes beyond the threshold value or the point crosses the boundary of the patch, the coordinate in the last frame will be used without any update. On the contrary, if the point meets the requirements, it will use the new position and the comparison will be updated in real-time.

### 2.2. Location Constraint

As the kernel of the algorithm, the classifier is the key factor that determines the tracking effect. How to generate samples and how to label them can have a direct impact on the quality of the classifier. Most algorithms [11,12,17] generate the training samples from the current frame after getting the new target location. By using the sampler and the labeler, samples can be generated in real-time. The sampler draws samples from the surrounding of the target. Then labeler will choose labels for these training samples and assign weights to them. Finally, the classifier will be updated by means of these training samples and labels. Throughout this process, positive samples and negative samples are always labeled in intuition rather than in an objective method, which will bring the label noise [20] into the classifier and influence the tracking effect. In addition, the weights are always assigned equally, meaning that a positive sample which overlaps observably with the tracking bounding box is regarded the same as the sample which overlaps a little. This slight inaccuracy will reduce the accuracy of the classifier and lead to further inaccuracy.

To solve these problems, we propose the location constraint and combine it with the learning part. We divide the region around the target in last frame into scanning grids. According to the coordinates of the grids, we assign weights and labels to the samples by means of the location constraint function.

#### 2.2.1. Scanning Grids

**Figure 4:**
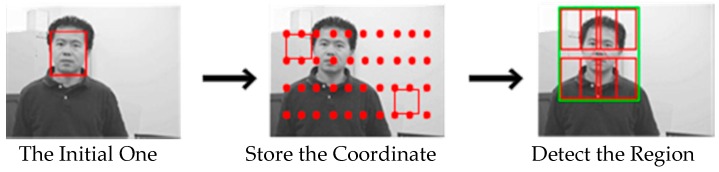
Brief process of generating the scanning grids and the region for detecting.

By using the spatio-temporal relationships, we generate all possible scanning grids around the initial bounding box with the following parameters: horizontal step=2 pixels, vertical step=2 pixels, bounding box size is equal to the initial box, and store the coordinates of each box’s upper left corner. This setting will produce around 80,000 bounding boxes for a PAL image (720×576) if the initial box size is 80×80 pixels. When considering the problem of real-time, we only detect boxes around the initial one in the detecting region, which is two times bigger than the bounding box size. This brief process is shown in Figure 4.

Although the computational complexity and the time consumption can be obviously decreased in the detecting region, the tracking bounding box will be lost if the target is occluded completely for a short while. To solve the problem, we adopt the global detection which can regain the target. We divide the frame into four blocks and detect each block in four consecutive frames. The feature vectors of each bounding box in the block are detected by means of the ensemble classifier till the target is regained.

For dealing the size variation of the object, we adopt the affine feature extraction. We generate the pixel comparisons randomly according to the bounding boxes whose sizes are from 40×40 to 120×120 pixels, and store them offline. When we get the target, we can also get the posterior of four bounding boxes (introduced in Section 2.2.2), whose sizes are bigger or smaller than the tracking bounding box. For example, if the tracking box size is 80×80 pixels, we can get the posterior values of the bounding boxes whose sizes are 82×82, 84×84, 78×78 and 76×76 pixels. At the same time the NCC value of each box will also be obtained. Finally, we can confirm the box size in next frame by calculating the maximum sum of the NCC and posterior value of each bounding box.

#### 2.2.2. Location Constraint

**Figure 5:**
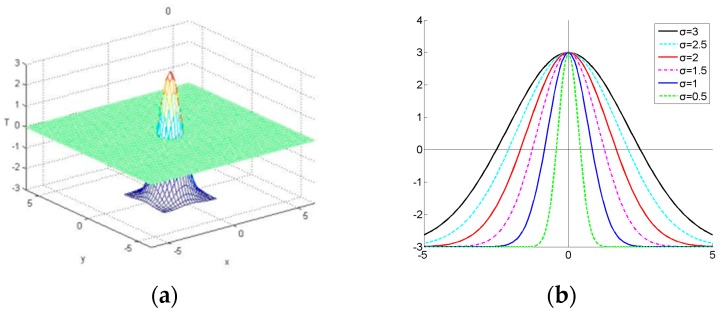
Simulation of the location constraint function: (**a**) display in three-dimensional; and (**b**) illustration of 2-D cross section of the location constraint function with different shape parameters σ.

The location constraint is inspired by [21]. It is a Gaussian fashion function, which labels the samples and distributes the weight objectively to each sample according to the location. The model is:

(1) $$\omega (x,y)=a\times \exp(-\left(x^{2}+y^{2}\right)/\sigma^{2})-a/2$$

where σ is the shape parameter, x and y is the coordinate of the bounding box, and a is the constant to control the weight. By adopting different constant a, we can control the maximum and minimum value of the weight and make the learning much faster. Through a number of experimental tests, we find that a small constant value (e.g., a = 1) may make the learning slow and a large value (e.g., a = 10) may lead to over learning. Thus, we find that a=6 can make the learning robust in our experiments. The simulation of the function can be seen in Figure 5a.

By utilizing different shapes of parameter σ, we can change the quantity of positive and negative samples. As illustrated in Figure 5b, a large σ (e.g., σ = 3) may generate excessive positive samples and result in an over-smoothing effect. On the other hand, a small σ (e.g., σ = 0.5) may generate a sharp peak close to the center of the target, and lead to over-fitting in search of the location of the target in the coming frame. Through the experiments, we find that the robust results can be obtained when σ = 2. Thus, when we get the target’s location, we can utilize the bounding boxes in the detected region and the location constraint to get the positive or negative samples accurately in real-time. Location constraint can not only resolve the label noise, but also improves the quality of learning. 

When we get the weight of each positive or negative sample, the posterior probability of each feature can also be obtained as follows:

(2) $$P(f)=\frac{\sum_{i=1}^{t}{W_{i}}^{+}(f)}{\sum_{i=1}^{t}{W_{i}}^{+}(f)+\sum_{i=1}^{t}{W_{i}}^{-}(f)}$$

where $\sum_{i=1}^{t}{W_{i}}^{+}(f)$ and $\sum_{i=1}^{t}{W_{i}}^{-}(f)$ represents the sum of the weights for the positive and negative samples from the first frame to frame t, f means the feature which is extracted in each fern. In Equation (2), ${W_{i}}^{+}(f)$ and ${W_{i}}^{-}(f)$ can be modeled as:

(3) $${W_{i}}^{+}(f)=\sum \omega (p^{+})$$

(4) $${W_{i}}^{-}(f)=\sum \left|\omega (p^{-})\right|$$

$\omega (p^{+})$ and $\omega (p^{-})$ in the Equations (3) and (4) are the weight variables which can be obtained from Equation (1). $p^{+}$ and $p^{-}$ can be defined as:

(5) $$p^{+}=\left\{(x,y)|(x,y)\in \Omega ,Ext(x,y)=f,\omega (x,y)\geq 0\right\}$$

(6) $$p^{-}=\left\{(x,y)|(x,y)\in \Omega ,Ext(x,y)=f,\omega (x,y)<0\right\}$$

Ext(x,y) means the process of the feature extraction in each fern. If the samples in different positions have the same feature, the related weights will be accumulated.

### 2.3. Combination and Learning

After filtering the patches by the ensemble classifier, we have several bounding boxes left which are supposed to include the target and treat them as promising boxes. To confirm the target from the patches which have been detected by the ensemble classifier, we adopt the combiner to assist in learning. The combiner is defined as an estimator which can combine the output of the ensemble classifier, location-weighted function and the online templates to confirm the target.

#### 2.3.1. Output of the Ensemble Classifier

By utilizing the location constraint, we can get the posterior probabilities of the features. Thus, in the next frame, the bounding boxes in the detecting region will be detected in real-time. Each bounding box will get its respective value by calculating as the following:

(7) $$p(f_{1},f_{2},...,f_{j},...,f_{n}|box)=\frac{1}{n}\sum_{j=1}^{n}P(f_{j})$$

where n is the count of the ferns and $f_{j}$ is the feature extracted from the bounding box. If the $p(f_{1},f_{2},...,f_{j},...,f_{n}|box)$ is greater than the threshold, it will be regarded as the promising bounding box. Otherwise, it will be excluded.

In this way, we can get all the promising boxes in the detecting region and define them as $\omega_{ferns}=\left\{p(f_{1},...f_{n}|box_{1}),p(f_{1},...f_{n}|box_{2}),...,p(f_{1},...f_{n}|box_{m})\right\}$, m is the total quantity of the promising boxes. Thus, each box will have its own value.

#### 2.3.2. Location-Weighted Function

To consider the spatio-temporal relationships, the target in the current frame can be expected to exist around the target’s position which is the center of the detecting region in the last frame. Thus, we construct the location-weighted function which has high weightings around the center and low weightings on the edge of the detecting region.

The location-weighted function is inspired by the Hanning window function [22], which is defined as:

(8) $$\omega (x)=0.5\left[1-\cos\left(\frac{2\pi x}{A+1}\right)\right],1\leq x\leq A$$

**Figure 6:**
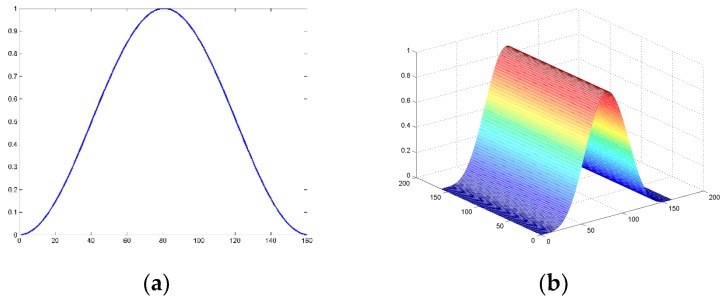
Simulation of the function: (**a**) Hanning window function; and (**b**) location-weighted function.

It is a cosine window function, and is show in Figure 6a.

Referring to this function, we construct the location-weighted function as follows and its 3-D simulation is shown in Figure 6b:

(9) $$w_{L}(x,y)=w_{han}(x){w_{han}}^{T}(y)$$

where the $\omega_{han}(x)$ and ${\omega_{han}}^{T}(y)$ is defined as:

(10) $$w_{han}(x)=0.5\left[1-\cos\left(\frac{2\pi x}{x_{\max}+1}\right)\right]$$

(11) $${w_{han}}^{T}(y)=0.5{\left[1-\cos\left(\frac{2\pi y}{y_{\max}+1}\right)\right]}^{T}$$

vector x and vector y are the coordinate vectors which can be represented as $x=\left\{x_{1},x_{2},...,x_{\max}\right\}$, $y=\left\{y_{1},y_{2},...,y_{\max}\right\}$. $\left\{x_{1},x_{2},...,x_{\max}\right\}$ and $\left\{y_{1},y_{2},...,y_{\max}\right\}$ are the coordinates of each promising box’s upper left corner relative to the left corner of the detecting region. Thus, each promising box will get the weight according to its position.

#### 2.3.3. Online Templates

**Figure 7:**
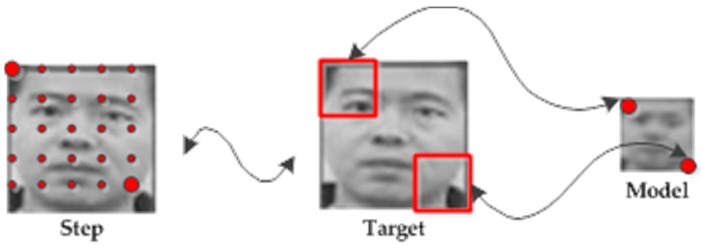
Brief process of the mean sampling.

The online templates can be regarded as the NN classifier in [12]. Taking time consumption and computational complexity into consideration, we compress the target bounding box into the model whose size is only 15×15 by means of the mean sampling (Figure 7) method. The promising box is then matched with the compressed templates by using normalized cross-correlation (NCC) [23].

The mean sampling method is a reliable and real-time sampling method. Although some information will be lost during runtime, the main characteristics will be maintained well. From Figure 7, we can get a brief process of mean sampling. Firstly, we can ensure the steps according to the ratio between the promising box and the model. Then, starting from the upper left corner of the promising box, we generate a sampling patch in each step point and the size of the sampling patch is a square whose side length is the same as the step (the vertical step or the horizontal step). In this way, we obtain a number of patches whose upper left corner is the step point. Next, we calculate the mean of the pixel values of each patch as follows:

(12) $$M_{mean}=\frac{1}{N\times N}\sum_{x=1}^{N}\sum_{y=1}^{N}pixel(x,y)$$

where N is the size of the sampling patch, pixel(x,y) is the value of the pixel in position (x, y). Finally, we can get $M_{mean}$ as the pixel of the model.

In this paper, the online templates are constructed by the compressive models and can be defined as $\left\{T_{1}(x,y),T_{2}(x,y),...,T_{K}(x,y)\right\}$, and K is the quantity of the models. Because of the limitation of memory space, we will only store fifty models.

After compressing the promising box, we match it with the models by means of normalized cross-correlation (NCC) as follows:

(13) $$\sigma =\frac{\sum_{x=1}^{M}\sum_{y=1}^{M}\left|S_{m}(x,y)-\bar{S_{m}}(x,y)\right|\left|T_{k}(x,y)-\bar{T_{k}}(x,y)\right|}{\sqrt{\sum_{x=1}^{M}\sum_{y=1}^{M}{\left(S_{m}(x,y)-\bar{S_{m}}(x,y)\right)}^{2}}\sqrt{\sum_{x=1}^{M}\sum_{y=1}^{M}{\left(T_{k}(x,y)-\bar{T_{k}}(x,y)\right)}^{2}}}$$

where the $S_{m}(x,y)$ is the compressive patches of the promising box m, the $T_{k}(x,y)$ is the model k which belongs to the online templates and M is the size of the models. The $\bar{S_{m}}(x,y)$ and the $\bar{T_{k}}(x,y)$ can be calculated by using the equation:

(14) $$\bar{S_{m}}(x,y)=\frac{1}{M\times M}\sum_{x=1}^{M}\sum_{y=1}^{M}S_{m}(x,y)$$

(15) $$\bar{T_{k}}(x,y)=\frac{1}{M\times M}\sum_{x=1}^{M}\sum_{y=1}^{M}T_{k}(x,y)$$

By using Equation (13), each promising box can get numbers of NCC values relative to the online templates. Furthermore, we can obtain the expectation of the NCC values for each promising box by

(16) $$\omega_{ncc}=E\left(\sigma \right)=\frac{1}{K}\sum_{k=1}^{K}\sigma_{k}$$

Here we define the output of the NCC as $\omega_{NCC}=\left\{\omega_{ncc}(box_{1}),\omega_{ncc}(box_{2}),...,\omega_{ncc}(box_{m})\right\}$. We also record each promising box’s minimum value of σ and define it as $\sigma_{\min}$. Thus, when we confirm the target, we can use the current compressive model to replace the model which has the minimum normalized cross-correlation value.

#### 2.3.4. Combination and Learning

Combination is used to confirm the target. It can also be regarded as the most important part of the learning. By uniting the output of the ensemble classifier $\omega_{ferns}$, location-weighted $w_{L}(x,y)$ and $\omega_{NCC}$, each promising box can be evaluated by the combiner. Thus, the classifier will be updated accurately in runtime.

As shown in Equation (17):

(17) $$box_{\max}=\arg\;\max\left[\omega_{ferns}(box_{i})*w_{L}(box_{i}(x,y))*\omega_{NCC}(box_{i})\right]$$

$box_{i}$ can be obtained from the promising boxes sequence $\left\{box_{1},box_{2},...box_{i},...,box_{m}\right\}$ $w_{L}(box_{i}(x,y))$ can be obtained by using the coordinate (x,y) of $box_{i}$ and the vector $w_{L}(x,y)$ in Equation (9). Thus, the promising box with a maximum value will be the target in the current frame. 

Once we confirm the target, we can utilize the target bounding box and the location constraint to get the positive and negative samples for ensemble classifier to learn. If its expectation of the NCC value is greater than the threshold (we observed that the best performance is in the range 0.75–0.85), the target model will be added to the online templates. If the quantity of the online templates falls out of the range, it will replace the model with the minimum normalized cross-correlation value

## 3. The Embedded System

**Figure 8:**
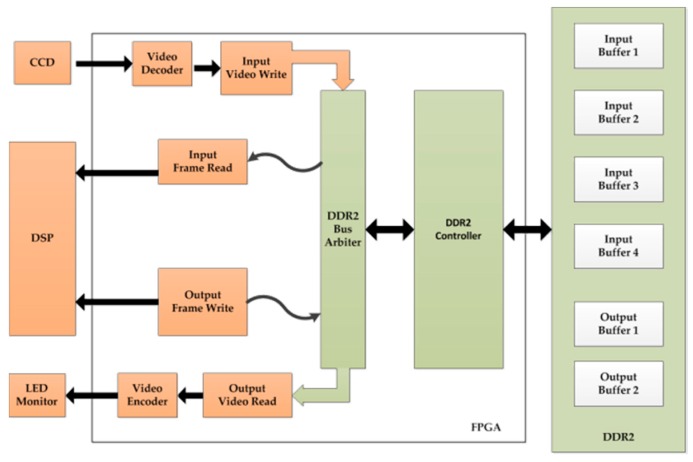
Underlying data flow architecture.

Figure 8 shows the underlying data flow architecture of the embedded system. It mainly includes three parts: image capture, image processing and image output. Image capture is completed by the CCD camera. The CCD camera used is a Mintron MTC-62V1 (720×576, PAL, Mintron Enterprise Co., Led., Taipei, China). A Computar focus lens (CBC Group Co., Ltd., Beijing, China) with a focal length in the range of 3.5–8 mm is used. Image processing is the most important part of the system and is implemented by DSP and FPGA unless otherwise stated. The DSP used is TMS320C6416 (TEXAS Instruments, Dallas, TX, USA), which has a 600 MHz clock rate and 4800 MIPS processing ability. This is used in the implementation of the AFCL framework and for communication with the servo system. The FPGA Cyclone III EP3C120F780 (Altera Company, Silicon Valley, CA, USA) is used for image preprocessing and main logic control. This is also connected to DDR2 memory to complete the real-time storage of the data. An 8” ZGYNK TFT-LED monitor (ZGYNK, Shenzhen, China) is utilized as the image output of the system. Its resolution is 1024×768, and the applicable power is 12 V.

## 4. Experimental Validation

### 4.1. Software Experiment

#### 4.1.1. Experimental Setup

The proposed framework is implemented on VS2010, Intel(R) Core(TM) i5-4570, 3.20 GHz CPU, 4 GB RAM, Win7 x86 system, in Beijing, China. We generate all possible scanning grids with the following parameters: horizontal step=2 pixels, and vertical step=2 pixels. The parameters of the location constraint in Equation (1) are: a=6, and σ=2. The ensemble classifier is constructed by 10 ferns, and each fern has 10 pairs of feature points we use to generate feature vectors. The size of the online templates is set to 50, and the model size is 15×15.

To verify whether the AFCL framework can handle the tracking problems well, including illumination variation, partial occlusion, object rotation, pose variation, background clutter, background noise, and so on, we evaluate the proposed framework on the benchmark data set [2] which includes 50 sequences and the outputs of 29 trackers. According to different challenging factors, the 50 sequences can be tagged with 11 attributes. To evaluate the trackers equally, we use the precision plot and the success plot. The precision plot can represent the percentage of frames, where the distance between the predicted bounding box location and the ground truth output is within a given threshold, whereas the success plot represents the percentage of frames, where the overlap ratio between the predicted bounding box and the ground truth output is higher than the given threshold t∈[0,1]. We show the results of one pass evaluation (OPE) [2] for the top 10 algorithms, including AFCL, TLD [12], Struck [20], OAB [24], SCM [25], ASLA [26], CXT [27], VTD [28], VTS [29], and CSK [30].

#### 4.1.2. Results

**Figure 9:**
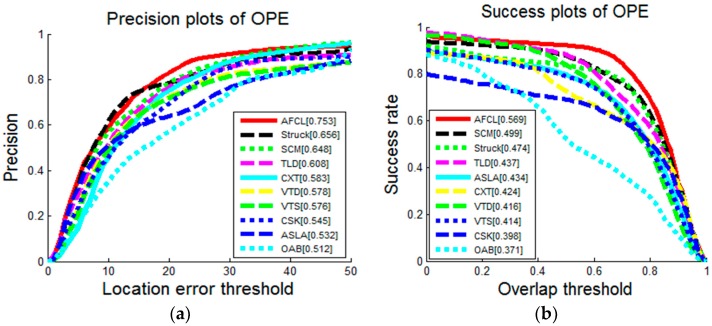
The precision plots and the success plots of OPE for top 10 trackers. Each tracker is ranked by the performance score. In the precision plot, the score is at error threshold of 20 pixels. In the success plot, the score is the AUC value.

The results of the precision plots and the success plots which compare the trackers on 50 sequences are shown in Figure 9. It is obviously that the proposed AFCL framework can perform better than the other trackers. For further analyses on the tracking performance, we also show the results on sequences with 11 attributes in Table 1 and Table 2.

From Table 1 and Table 2, we can note that the AFCL framework can outperform other state-of-the-art methods in the challenge of IV, SV, OCC, DEF and IPR. Although the proposed framework can perform well in some conditions, the great failure should not be neglected in the challenge of LR and OV. Due to the pixel comparisons, the feature vectors will be influenced in the low resolution condition. It will bring the noise to the classifier and lead to the failure of tracking. In our method, we take the spatio-temporal relationships into account. We ensure the detecting region around the target, which has been found in previous frame. Thus, when the target is out of view, a region may be chosen in the background resulting in tracking failure.

To validate the improvement on computational complexity, we show the average fps of the top 10 algorithms in Table 3.

Although the AFCL framework is not the fastest, it can implement the tracking with 30.2 fps on average. By using the spatio-temporal relationships, we can decrease the computational complexity and the time consumption in the detecting region. Using the random ferns as the basic classifier can also improve the efficiency during runtime. In consideration of the memory space and the computational complexity, we use mean sampling to compress the bounding box. Thus, the similarity calculation can be implemented in real-time.

**Figure 10:**
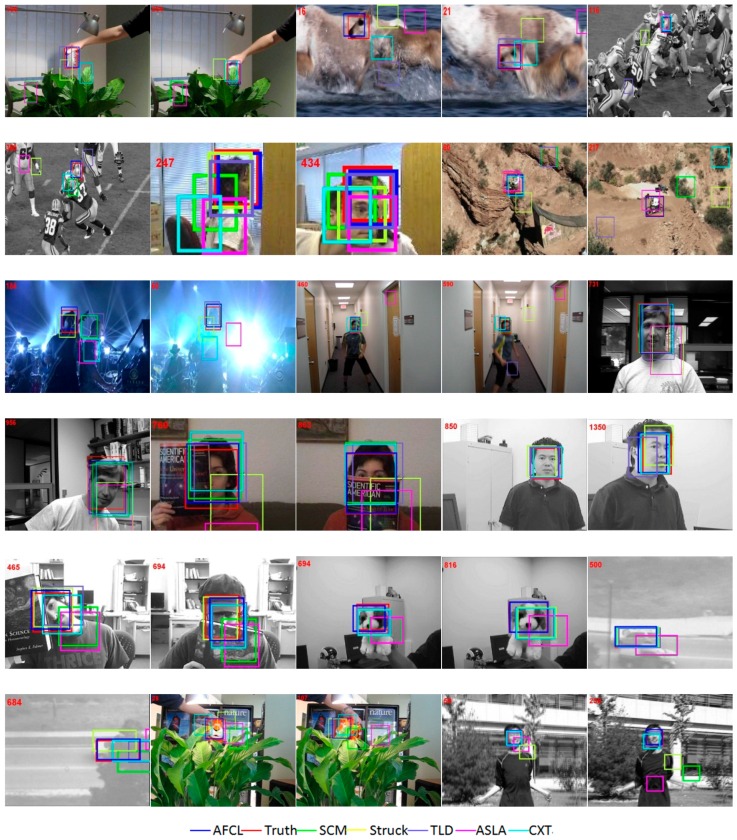
Tracking results of the sequences.

The tracking results of the sequences contain the typical challenges are also shown in Figure 10. For clarity, we only mark the top six results and the ground truth position in each figure. On the one hand, due to adaptive feature vectors, the characteristics of the object can also be described accurately even if the target rotates or varies (e.g., Coke, Girl, Boy, etc.). When the background becomes cluttered or appears with illumination variations (e.g., Shaking, Mhyang, Coke, etc.), the features can be adjusted in real-time and maintain their accuracy by means of the F–B error. On the other hand, unlike other methods, the samples that are used to learn are generated by location constraints. It can not only decrease the label noise, but also addresses the drifting problem and impairs the sampling influence in follow-up tracking conditions. Thus, even facing the partial occlusion (e.g., FaceOcc1, FaceOcc2, Tiger1, Suv, etc.), the framework can also complete the tracking accurately. Compared with other approaches, we use the combiner as an important part of learning and make the ensemble classifier updatable during runtime. By using the combiner, the learning process will be further refined even if the tracking conditions become more challenging (e.g., Football, Deer, Mountainbike, Jumping, etc.). The online templates can revise the noise from the classifier, and supervise the quality of the learning during runtime as well.

### 4.2. Hardware Experiment

#### 4.2.1. Real-Time Tracking System

**Figure 11:**
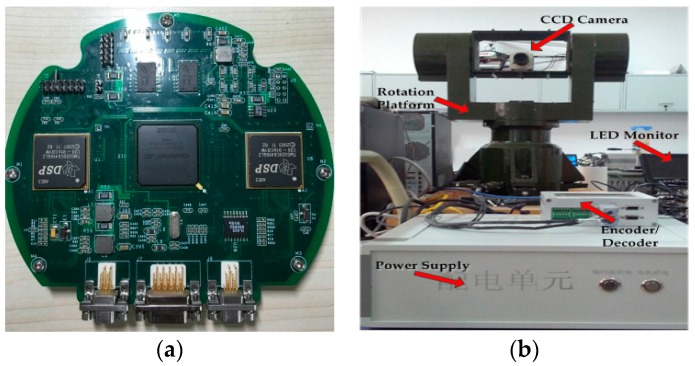
Real-Time tracking system: (**a**) embedded system; and (**b**) servo system.

Within this work, we set up a real-time tracking system based on an AFCL framework (see in Figure 11). This is composed of the embedded system with TMS320C6416 and Altera Cyclone III as the kernel processors, the servo system contains the CCD camera, LED monitor and the intelligent rotation platform. The parameters of the embedded system, the CCD camera and the LED monitor are introduced in Section 3. The intelligent rotation platform is produced by Changchun Institute of Optics, Fine Mechanics and Physics, Chinese Academy of Science. It can communicate with the embedded system by means of the encoder/decoder module, which has the RS-232 connector. We use a power supply module to convert 220 V AC into 12 V DC, and provide power for the platform, CCD camera, LED monitor and the embedded system. The first two hundred frames are used as the preparation stage, which can then adjust the platform and confirm the tracking target. Furthermore, the framework is implemented on the embedded system by means of CCS 3.3.

#### 4.2.2. Experimental Results

**Figure 12:**
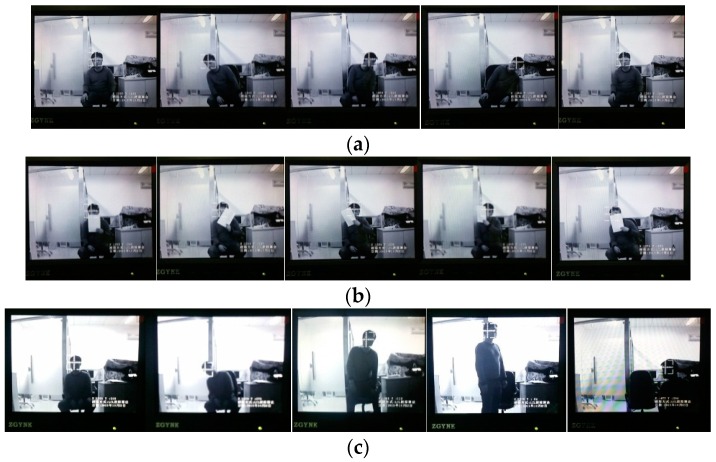
Experimental results of the real-time tracking system: (**a**) pose variation; (**b**) partial occlusion; and (**c**) illumination variation.

The results of our experiments are shown in Figure 12. These were performed within the key Laboratory of Photoelectronic Imaging Technology and System, Ministry of Education of China, Beijing. Here, the sequence (a)–(c) is captured during a ten minute runtime.

From the figure, it is clear that the tracking effect is stable when the target has pose variation, partial occlusion and illumination variation. This effectively validates the analysis in software experiments and tests the feasibility of the proposed framework. In addition, the tracking system can run at the rate of 25 fps and maintains accuracy during runtime without the phenomenon of frame loss.

## 5. Conclusions

In this paper, we propose a novel tracking method based on an AFCL framework to construct a real-time tracking system. To solve the challenging problems in the tracking process, we make the following principal contributions: Firstly, the adaptive features are adopted to make the tracking more robust and stable by utilizing the Forward–Backward error and L–K tracking to update the pixel pairs. Thus, each fern can be adaptive during runtime and thereby effectively avoids the deviation caused by object noise. Secondly, the location constraint is proposed to decrease label noise. Therefore, we can assign the weight to each sample according to its location. In addition, all the positive or negative samples can be treated as uncoordinated samples according to their weights. Thirdly, we use a combiner to assist the learning process. By combining the outputs from the NCC, the posterior probability and the value from the location-weighted function, the target boundary box can be confirmed in real time. As a result, evaluation of the widely used tracking benchmark [2] shows that the proposed framework can significantly improve the tracking accuracy, and decrease the processing time. Finally, the experiments of the tracking system that is composed of the embedded system and the servo system demonstrate the efficiency and robustness of the AFCL framework comprehensively.

## Figures and Tables

**Table 1:** Average success scores on different attributes: IV (illumination variation), OPR (out-of-plane rotation), SV (scale variation), OCC (occlusion), DEF (deformation), MB (motion blur), FM (fast motion), IPR (in-plane rotation), OV (out-of-view), BC (background cluttered) and LR (low resolution). The best results are in red and the second best results are in green.

	SCM	Struck	TLD	ASLA	CXT	VTD	VTS	CSK	OAB	AFCL
IV	0.473	0.428	0.402	0.429	0.368	0.420	0.428	0.369	0.302	0.639
OPR	0.470	0.432	0.423	0.422	0.418	0.435	0.425	0.386	0.359	0.451
SV	0.518	0.425	0.424	0.452	0.389	0.405	0.400	0.350	0.370	0.629
OCC	0.487	0.413	0.405	0.376	0.372	0.404	0.398	0.365	0.370	0.636
DEF	0.448	0.393	0.381	0.372	0.324	0.377	0.368	0.343	0.351	0.562
MB	0.293	0.433	0.407	0.258	0.369	0.309	0.304	0.305	0.324	0.424
FM	0.296	0.462	0.420	0.247	0.388	0.303	0.299	0.316	0.362	0.558
IPR	0.458	0.444	0.419	0.425	0.452	0.430	0.415	0.399	0.347	0.644
OV	0.361	0.459	0.460	0.312	0.427	0.446	0.443	0.349	0.414	0.333
BC	0.450	0.458	0.348	0.408	0.338	0.425	0.428	0.421	0.341	0.452
LR	0.279	0.372	0.312	0.157	0.312	0.177	0.168	0.350	0.304	0.286
Overall	0.499	0.474	0.437	0.434	0.424	0.416	0.414	0.398	0.371	0.569

**Table 2:** Average precision scores on different attributes: IV (illumination variation), OPR (out-of-plane rotation), SV (scale variation), OCC (occlusion), DEF (deformation), MB (motion blur), FM (fast motion), IPR (in-plane rotation), OV (out-of-view), BC (background cluttered) and LR (low resolution). The best results are in red and the second best results are in green.

	SCM	Struck	TLD	ASLA	CXT	VTD	VTS	CSK	OAB	AFCL
IV	0.594	0.558	0.537	0.517	0.501	0.557	0.572	0.481	0.398	0.827
OPR	0.618	0.597	0.596	0.518	0.574	0.620	0.603	0.540	0.510	0.592
SV	0.672	0.639	0.606	0.552	0.550	0.597	0.582	0.503	0.541	0.798
OCC	0.640	0.564	0.563	0.460	0.491	0.546	0.533	0.500	0.492	0.823
DEF	0.586	0.521	0.512	0.445	0.422	0.501	0.487	0.476	0.470	0.532
MB	0.339	0.551	0.518	0.278	0.509	0.375	0.375	0.342	0.360	0.550
FM	0.333	0.604	0.551	0.253	0.515	0.353	0.351	0.381	0.431	0.761
IPR	0.597	0.617	0.584	0.511	0.610	0.600	0.578	0.547	0.479	0.832
OV	0.429	0.539	0.576	0.333	0.510	0.462	0.455	0.379	0.454	0.501
BC	0.578	0.585	0.428	0.496	0.443	0.571	0.578	0.585	0.446	0.786
LR	0.305	0.545	0.349	0.156	0.371	0.168	0.187	0.411	0.376	0.423
Overall	0.648	0.656	0.608	0.532	0.583	0.578	0.576	0.545	0.512	0.753

**Table 3:** Speeds and implementations of the tracking methods. fps, frames per second the best results are in red and the second best results are in green.

Tracker	SCM	Struck	TLD	ASLA	CXT	VTD	VTS	CSK	OAB	AFCL
Average fps	0.51	20.2	28.1	8.5	15.3	5.7	5.7	362	22.4	30.2
